# 肺部磨玻璃结节的诊治策略

**DOI:** 10.3779/j.issn.1009-3419.2018.03.06

**Published:** 2018-03-20

**Authors:** 群 王

**Affiliations:** 200032 上海，复旦大学附属中山医院胸外科 Department of Toracic Surgery, Zhongshan Hospital, Fudan University, Shanghai 200032, China

**Keywords:** 肺肿瘤, 肺部磨玻璃结节, 诊断, Lung neoplasms, Pulmonary ground glass nodule, Diagnosis

## Abstract

肺部磨玻璃结节（ground glass nodule, GGN）是一种影像学表现，可能是肺部恶性肿瘤或良性病变。目前对于肺部磨玻璃结节的诊疗仍存在争议。2017年Fleischner协会和美国国立综合癌症网络（National Comprehensive Cancer Network, NCCN）都更新了GGN诊疗的指南，与之前的版本相比，手术或活检的指征更严，随访的间隔时间更长。临床工作中，GGN的大小、实性成分大小、动态随访变化和CT值都是判断手术介入时机的因素。GGN的诊疗中还存在一些误区：抗生素的使用、正电子发射型计算机断层显像（positron emission tomographycomputed tomography, PET-CT）检查、贴近胸膜的纯GGN和进入GGN的血管都是值得注意的问题。总之，GGN是一种发展缓慢的病灶，可以安全地进行随访。

## Abstract (en)

Pulmonary ground glass nodule (GGN) is a term of radiological manifestation, which may be malignant or benign. Te management for pulmonary GGN remains controversial. Both Fleischner society and National Comprehensive Cancer Network (NCCN) panel updated the guideline for the management of GGN in 2017. Compared with previous versions, the indication for surgery or biopsy is stricter, and the recommended follow-up interval is prolonged. In clinical practice, the size of GGN component, the size of consolidation component, dynamic change during follow-up and computed tomography (CT) value are the four factors that help surgeons to decide the timing of surgery. Tere are some misunderstandings for the management of GGN, such as the administration of antibiotics, the use of positron emission tomography-computed tomography (PET-CT), pure GGN adjacent to visceral pleura, and GGN with penetrating vessel. In conclusion, GGN is a kind of slowly growing lesion, which can be followed up safely.

肺部磨玻璃结节(ground glass nodule, GGN)是指计算机断层扫描(computed tomography, CT)上边界清楚或不清楚的肺内密度增高影，其病变密度不足以掩盖其中走行的血管和支气管影。GGN可能是恶性肿瘤、良性肿瘤、炎症、肺间质性疾病或肺内淋巴结等等。GGN的病理基础是肺泡隔增厚或部分肺泡腔充满液体、细胞或组织碎片^[1]^。1995年日本的Noguchi提出了肺腺癌的野口分型，到2011年国际肺癌研究协会/美国胸科学会/欧洲呼吸学会(International Association for the Study of Lung Cancer/American Thoracic Society/European Respiratory Society, IASLC/ATS/ERS)发布了肺腺癌的新分型^[2]^，腺癌的不同类型有不一样的影像学表现。

Fleischner协会2013年提出了磨玻璃结节的诊疗指南^[3]^。对于≤5 mm的纯GGN不需要随访，对于> 5 mm的纯GGN应在3个月后复查CT，如果GGN仍然存在且没有变化，则每年CT随访，至少持续3年。对于部分实性结节，应在3个月后复查CT，如果仍然存在，且实性成分 < 5 mm，则每年CT随访，至少持续3年；如果实性成分≥5 mm，则推荐活检或手术治疗。2017年Fleischner协会更新了指南^[4]^，新指南将5 mm的临界值提高为6 mm，且延长了随访间隔。新指南认为临床工作中， < 6 mm的结节很难判断是否存在实性成分，所以通常不需要随访。

2016年及之前的美国国立综合癌症网络(National Comprehensive Cancer Network, NCCN)肺癌筛查指南中，并未将纯GGN和部分实性GGN分开，以5 mm和10 mm为分界线，制定了不同的随访间隔。在2017年的NCCN肺癌筛查指南中，对于20 mm以下的纯GGN，建议每年随访，对于20 mm以上的纯GGN，建议6个月内行LDCT复查。20 mm以下的纯GGN即使随访中变大，也可以继续随访，只有> 20 mm的纯GGN随访中增大，才可以考虑活检或手术。对于部分实性GGN，以实性成分6 mm和8 mm为界，采取不用的随访策略。只有实性成分≥ 6 mm且高度怀疑肺癌时才建议行活检或手术切除。2017年NCCN首次提到了偶然发现肺结节者的诊疗流程。 < 5 mm的单发纯GGO无需随访，≥5 mm的单发纯GGO在3个月后随访CT，如果仍存在则每年随访CT并至少持续3年。对于部分实性GGN，如果实性成分 < 5 mm，则可以继续随访；如果实性成分≥5 mm，则可以考虑活检或手术切除。2013年的美国胸科医师学会(American College of Chest Physicians, ACCP)肺部结节评估指南^[5]^提出：对于≤5 mm的纯GGN，不建议进一步评估，对于> 5 mm的纯GGN，建议每年进行随访，至少持续3年；对于≤8 mm的部分实性结节，第3个月、12个月、24个月随访，随后1年-3年每年随访，对于> 8 mm的部分实性结节，第3个月复查CT，如果持续存在，可进一步检查[正电子发射型断层显像(positron emission tomography, PET-CT)、非手术活检或手术切除]。

对GGN的长期随访能够揭示GGN的生长变化规律。一项来自韩国的研究^[6]^，回顾了1997年-2006年韩国LDCT筛查的19, 919例病例，其中发现且随访超过2年持续存在的纯GGO共122个，经过59个月的中位随访期，90.2%的GGO没有变化或缩小，增大的GGO中位体积倍增时间769天。有11例接受了手术切除，术后病理为：2例原位腺癌(adenocarcinoma *in situ*, AIS)，6例微浸润腺癌(minimally invasive adenocarcinoma, MIA)，3例浸润性腺癌。日本国立癌中心牵头开展了一项多中心前瞻性研究^[7]^，入组标准为GGN≤3 cm，实性成分≤5 mm，研究平均随访时间为(4.3±2.5)年。研究将GGN分为三种类型：纯GGN、异质性GGN(仅肺窗可见实性成分)、部分实性GGN(纵隔窗可见实性成分)。入组时有1, 046例纯GGN，81例异质性GGN，102例部分实性GGN。1, 046例纯GGN种，13例(1.2%)发展为异质性GGN，56例(5.4%)发展为部分实性GGN，平均变化时间为(3.8± 2.0)年。81例异质性GGN中16例(19.8%)发展为部分实性GGN，平均变化时间为(2.1±2.3)年。研究还发现，纵隔窗上，MIA的实性成分最长径平均3.3 mm，浸润性腺癌为5.5 mm；1, 229枚GGN中，仅4.2%为MIA或浸润性腺癌；结节最大径是GGN生长的预测因素；只有部分实性GGN中才有浸润性肺癌。

GGN的大小、实性成分大小、动态随访变化和CT值都是我们判断手术介入时机的因素。Kim^[8]^回顾40例GGO，发现 < 5 mm的GGO均为良性，5 mm-10 mm的GGO中仅10.5%是恶性。作受试者工作特征曲线(receiver operating characteristics, ROC)分析后他认为8 mm可以作为GGO随访的临界值。多中心临床试验JCOG0201^[9]^入组了≤3 mm的外周型GGN 545例，最后发现 < 2 cm的外周型GGO且C/T值 < 25%时表现为为非侵袭性肿瘤(未侵犯淋巴结/血管/淋巴管)，随访后这部分患者的5年生存率97.1%。一项前瞻性研究^[10]^纳入了38例3 cm以下的GGO，患者进行高分辨率CT并行三维重建后计算GGO的平均CT值，手术后病理为10例不典型腺瘤样增生(atypical adenomatous hyperplasia, AAH)，21例细支气管肺泡癌，12例腺癌，计算发现平均CT值-472 HU是鉴别腺癌和细支气管肺泡癌的临界值。

临床工作中有一些5 mm-10 mm的GGN可以积极干预：(1)贴近脏层胸膜的外周型GGN，可局部切除；(2)存在高危因素：既往恶性肿瘤病史、家族史、长期吸烟史；(3)影像学存在恶性肿瘤征象：分叶征、毛刺征、胸膜凹陷、部分实性；(4) PET-CT代谢增高；(5)患者对于GGN极度焦虑，无法缓解。还有一些5 mm-10 mm的GGN需谨慎处理：(1) GGN位于肺实质内，无法局部切除；(2)未动态随访的纯GGO；(3)部分实性GGN，但影像学无恶性征象，或PET/CT表现为无代谢或低代谢；(4)高龄，一般状况差；(5)多发GGN。

GGN的诊疗过程中存在一些误区。(1)抗生素的应用。Fleischner指南指出对于GGN没有抗生素的指征。Khokhar^[11]^回顾了293例小结节，按随访期是否使用抗生素分为两组，结果发现小结节患者使用或不使用抗生素，结节缓解率为33%和27%，无明显差别。有肺部症状或CT有感染征象的亚组中，缓解率同样无明显差别。(2) PET-CT检查。Fleischner指南^[3]^认为对于小的纯GGN，PET-CT没有诊断价值，对于8 mm-10 mm的部分实性GGN，在进行创伤性的检查前建议进行PET-CT检查。(3) GGN贴近胸膜容易胸膜腔播散。有研究表明纯GGN不会侵犯脏层胸膜^[12]^，所以不会有胸膜腔播散的风险。(4)血管进入GGN是不良因素。肿瘤生长的血供主要来自体循环，而CT中看到进入GGN的血管来自肺循环，与肿瘤生长无关。目前并没有研究支持这一说法。

总而言之，GGN是一种发展缓慢的病灶，可以安全地随访。手术介入的时机有多种因素决定。抗生素、PET/CT对于纯GGO的价值有限。
